# Plasticizers Derived from Biomass Resources: A Short Review

**DOI:** 10.3390/polym10121303

**Published:** 2018-11-24

**Authors:** Puyou Jia, Haoyu Xia, Kehan Tang, Yonghong Zhou

**Affiliations:** 1 Institute of Chemical Industry of Forest Products, Chinese Academy of Forestry (CAF); Co-Innovation Center of Efficient Processing and Utilization of Forest Resources, Nanjing Forestry University; Key Lab of Biomass Energy and Materials, 16 Suojin North Road, Nanjing 210042, China; 2 College of Chemical Engineering, Nanjing Tech University, 30 Pu Zhu Road, Nanjing 211800, China; xiahaoyu0704@foxmail.com (H.X.); zdlzy5khan@163.com (K.T.)

**Keywords:** plasticizer, polyvinyl chloride, biomass resources, review

## Abstract

With rising environmental concerns and depletion of petrochemical resources, biomass-based chemicals have been paid more attention. Polyvinyl chloride (PVC) plasticizers derived from biomass resources (vegetable oil, cardanol, vegetable fatty acid, glycerol and citric acid) have been widely studied to replace petroleum-based *o*-phthalate plasticizers. These bio-based plasticizers mainly include epoxidized plasticizer, polyester plasticizer, macromolecular plasticizer, flame retardant plasticizer, citric acid ester plasticizer, glyceryl ester plasticizer and internal plasticizer. Bio-based plasticizers with the advantages of renewability, degradability, hypotoxicity, excellent solvent resistant extraction and plasticizing performances make them potential to replace *o*-phthalate plasticizers partially or totally. In this review, we classify different types of bio-based plasticizers according to their chemical structure and function, and highlight recent advances in multifunctional applications of bio-based plasticizers in PVC products. This study will increase the interest of researchers in bio-based plasticizers and the development of new ideas in this field.

## 1. Introduction

Plasticizers are among the most important additives required for the processing of polymer materials, especially polyvinyl chloride (PVC) plastics, which accounts for more than 60% of the total yield of plastic auxiliaries [1,2]. Traditional petroleum-based phthalate plasticizers are the most widely used globally. The yield and consumption of traditional phthalate plasticizers account for a large proportion of the total plasticizer production and sales, but they are gradually limited due to potential threats to human health and environment. Strict regulations on environmental protection and safety have been formulated and carried out. The development of environmentally-friendly non-toxic plasticizers and biodegradable bio-based plasticizers to replace phthalates has been a research hot spot. Non-toxic green plasticizers with high performance, oil resistance, extraction and migration resistance used in electrical insulation, food packaging, and medical and health products are constantly being developed, produced and applied. Plasticizers are functional additives, which are used to improve flexibility, plasticity, processability, and elongation of polymers, especially in PVC products [3]. When a plasticizer is added to a polymer, the intermolecular subvalent bond force is weakened, the crystallinity is lowered, the relative movement between the molecular segments is increased and the plasticity of the material is improved. Therefore, plasticizers are mainly used to decrease hardness, softening temperature, elastic modulus, and embrittlement temperature of polymers, while improving their flexibility and elongation. Non-phthalate plasticizer alternatives include citric acid ester, phosphates, polyesters, halogenated alkanes, and epoxy compounds [4,5,6,7]. With the increase in people’s awareness of environmental protection, especially the discovery of potential threats to human health by phthalate plasticizers and environmental pollution problems, the global hygienic requirements for plastic additives are increasing. The use of non-phthalate plasticizer alternatives has been gradually introduced [8,9,10].

## 2. Plasticizer Derived from Biomass Resources 

### 2.1. Biomass Based Epoxidized Plasticizer

Epoxidized plasticizer is a kind of environmentally-friendly plasticizer used in plastics industry, rubber industry, and coatings and new polymer materials [11,12]. Compared with other plasticizers, the epoxy group in the epoxidized plasticizer structure could absorb and neutralize the hydrogen chloride released by PVC during light or thermal degradation, which restricts or delays the continuous decomposition of PVC, endows PVC products with good light and thermal stability and extends their service life. Besides, it has been approved for food packaging and medical equipment materials in many countries and regions due to the extremely low toxicity of epoxidized plasticizers, which has made its production and price grow in recent years. Biomass-based epoxidized plasticizers mainly include epoxidized vegetable oils, epoxidized fatty acid esters and epoxy group containing cardanol derivatives. Currently, vegetable oils and epoxidized fatty acid esters have been used on the market [13,14].

**Figure 1:**
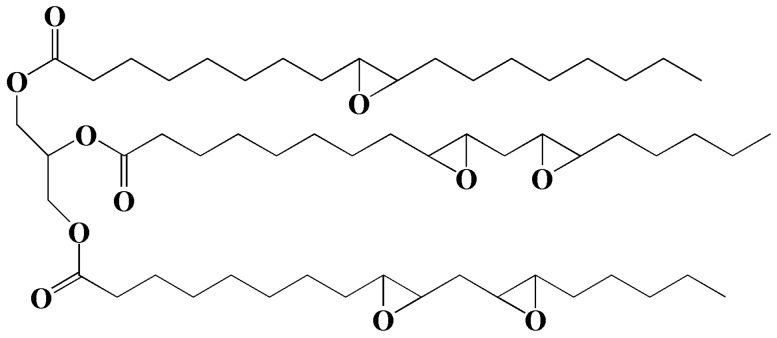
Chemical structure of epoxidized soybean oil.

Epoxidized soybean oil (ESO) is a collection of organic compounds obtained from the epoxidation of soybean oil that have been widely used as plasticizers and heat stabilizers of PVC materials. The chemical structure of ESO is shown in Figure 1. Ferrer et al. found that formulations based on PVC with different amounts of ESO (from 30 to 50 wt %) increased compatibility and improved thermal stability [15]. Park et al. investigated the performances of epoxy resins plasticized with ESO [16]. They found that the thermal stability and glass transition temperature of epoxy resins reduced with the addition of ESO. This phenomenon occurs due to the decreased density of the epoxy network. The addition of ESO causes the increase of stress intensity factor along with the flexural strength. Zhao et al. investigated the plasticizing effect of ESO on polybutylene succinate (PBS) [17]. The results show that ESO efficiently improves elongation at break of PBS. The elongation at break reaches a maximum value when the content of ESO is 5 wt % of PBS, 15 times more than the elongation of pure PBS. Moreover, ESO enhances the elongation at break of poly(lactic acid) (PLA) by 63%. Xu et al. studied the performances of PLA plasticized with ESO [18]. The results show that 9 wt % ESO increased the elongation at break of PLA by about 63%. PLA blends containing 6 wt % ESO show maximum tensile strength and melt strength. Zhu et al. added maleic anhydride (MA) to enhance PLA’s reactivity with ESO, obtaining an elongation at break of about 140% [19]. This can be explained by the grafting content of MA, which determines the properties of the blends. Xu et al. investigated the effect of ESO on melt rheological properties such as shear viscosity and melt strength of PLA blends [20]. They chose melt flow index (MFI) as measuring rule. The results show that PLA/ESO blends possess more MFI than pure PLA. Under the temperature range of 160–180 °C, PLA blends with 6 wt % ESO achieve the maximum melt strength. 

**Figure 2:**
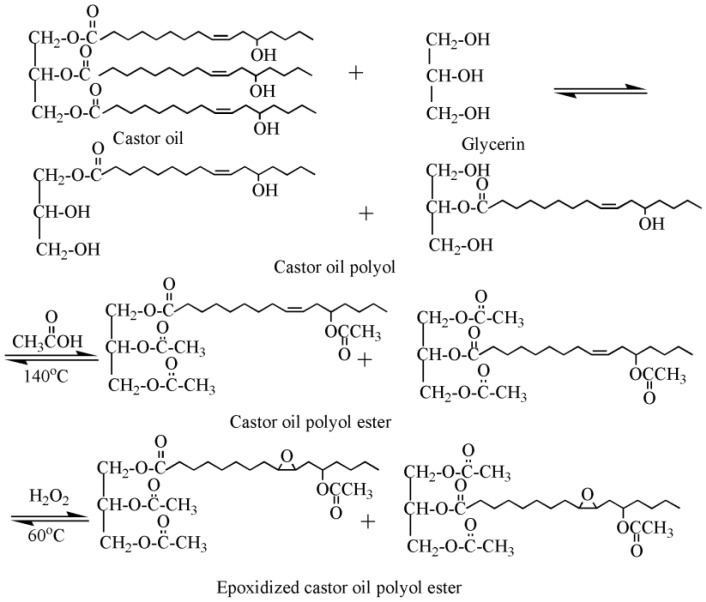
Synthesis of epoxidized castor oil polyol ester.

Our group synthesized a series of epoxidized castor oil polyol esters, epoxidized soybean oil polyol esters, and epoxidized tung oil methyl ester via alcoholysis and epoxidation, and investigated their plasticizing effect on PVC films [13,21,22,23]. Figure 2 shows the synthesis route of epoxidized castor oil polyol ester. Dynamic thermomechanical analysis (DMA) and differential scanning calorimeter (DSC) results show that these epoxidized vegetable polyol esters present excellent plasticizing effect on PVC and can be used as main plasticizers to completely replace DOP in flexible PVC films.

**Figure 3:**
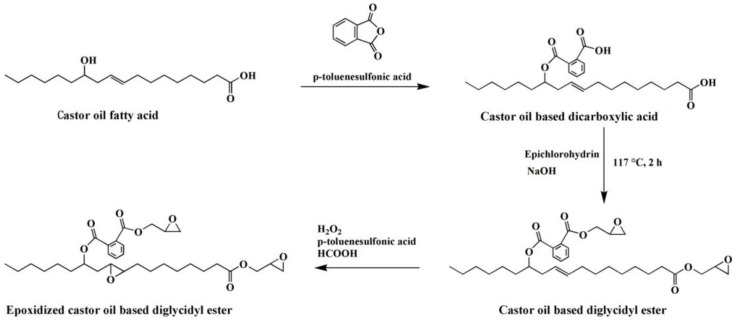
Synthesis of epoxidized castor oil based diglycidyl ester.

**Figure 4:**
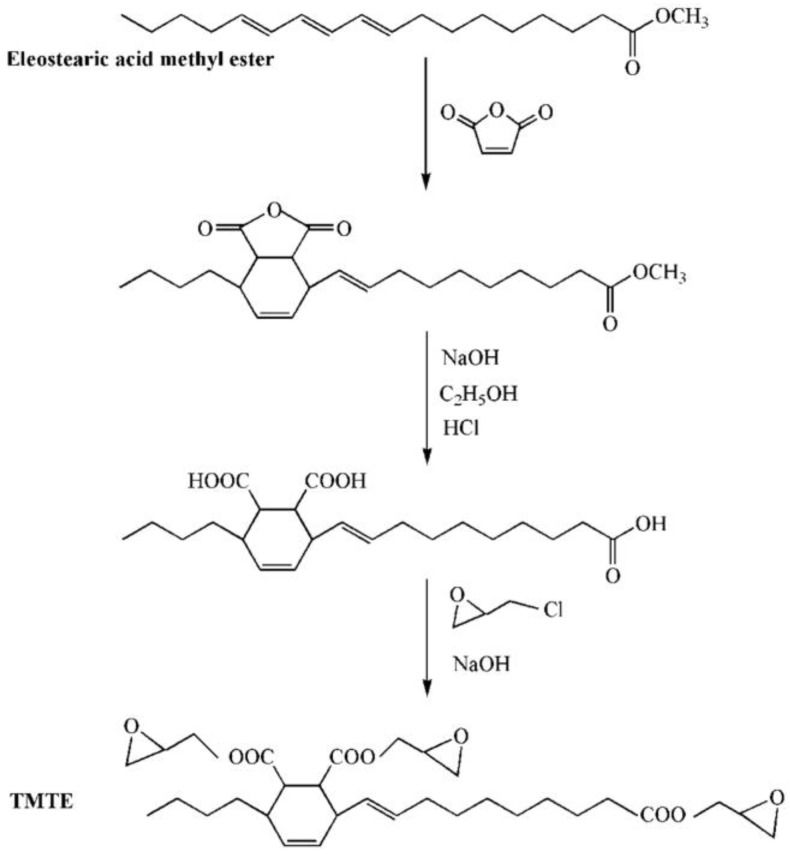
Synthesis of tung-maleic triglycidyl esters (TMTE).

Vieira et al. investigated the effect of polyesterification of rice fatty acid on properties of natural polymeric plasticizer with the usage of monopropylene glycol, octanol and diethylene glycol [24]. The experiment shows that elongation at break of PVC materials plasticized with natural polymeric plasticizer reaches 371.2%. To learn more about rice fatty acid, Machado et al. obtained two natural epoxidized plasticizers from peracetic acid (NP-Ac) and peroctanoic acid (NP-Oc). Even though both enhance the performance of PVC materials, natural epoxidized plasticizers derived from NP-Ac possess better ability than NP-Oc. This phenomenon may result from a higher degree of epoxidation in the process of reaction with acid [25]. Chaudhary et al. found that almost all kinds of epoxidized fatty acid esters (EFAE) are compatible with PVC and the efficiencies increase with decreasing molecular weight [26]. The alternatives achieve the balance of flexibility retention and mechanical properties after heat aging, especially compared with phthalate and trimellitate plasticizers. Espinosa et al. found that fatty acids obtained from rapeseed oil could serve as another cheaper raw source to produce plasticizers via thiol-ene addition and epoxidation reaction [27]. Silverajah et al. reported a comparative study on PLA/epoxidized palm oil blend about their mechanical and characterization [28]. They found that 1 wt % of epoxidized palm oil (EPO) is enough to enhance the flexibility and strength of PLA along with the flexural and impact properties. To obtain the best performance, EPO(3), a mixture of EPO and soybean oil is added. Pure PLA presents a tensile modulus value of 1054 MPa while that of EPO(3) is 1214 MPa. Mulla et al. studied the reaction between PLA and EPO by casting process at different weight ratio and found that the elongation at break reaches its best (about 210%) when the ratio of PLA/EPO blend is 80/20 [29]. Chieng et al. studied the cold-crystallization temperature when PLA is blended with EPO [30]. The results show that the mobility of the polymeric chains is enhanced. Furthermore, mild interfacial adhesion between PLA and EPO is obtained after adding 1 wt % epoxidized palm oil. Benzene groups containing bio-based plasticizer is also investigated. Chen et al. synthesized and used epoxidized castor oil based diglycidyl ester to replace DOP [31]. The synthesis route of epoxidized castor oil based diglycidyl ester is shown in Figure 3. The addition of epoxidized castor oil based diglycidyl ester significantly enhances thermal stability, compatibility, and flexibility of PVC films, presenting plasticizing effect and thermal stabilization of PVC films. PVC films plasticized with epoxidized castor oil based diglycidyl ester show good elongation at break (332.9%) and remarkable increase in flexibility and plasticization than DOP. In addition, the tung-maleic triglycidyl esters, which has similar chemical structure with epoxidized castor oil based diglycidyl, is also reported [32]. Figure 4 shows the chemical structure and synthesis route of epoxidized castor oil based diglycidyl, which shows similar plasticizing effect and thermal stabilization on soft PVC films. 

Epoxidized sunflower oil is well miscible with polylactic acid (PLA) and PVC [33,34]. Benaniba et al. studied the stabilizing effect of ESO on the thermal degradation of PVC [35]. The results suggest that ESO improves the thermal degradation of PVC. Atek et al. investigated the migration of epoxidized sunflower oil from plasticized PVC into food simulants [36]. Chromatography–mass spectrometry (GC-MS) is carried out to investigate the migration of ESO. Linoleic acid (C18:2) is used as external standard. The study provides new method to detect the loss of plasticizer in plasticized PVC materials. Lardjane et al. reported that both nature and the content of plasticizers acted as influential factors in the migration of epoxidized sunflower oil [37].

**Figure 5:**
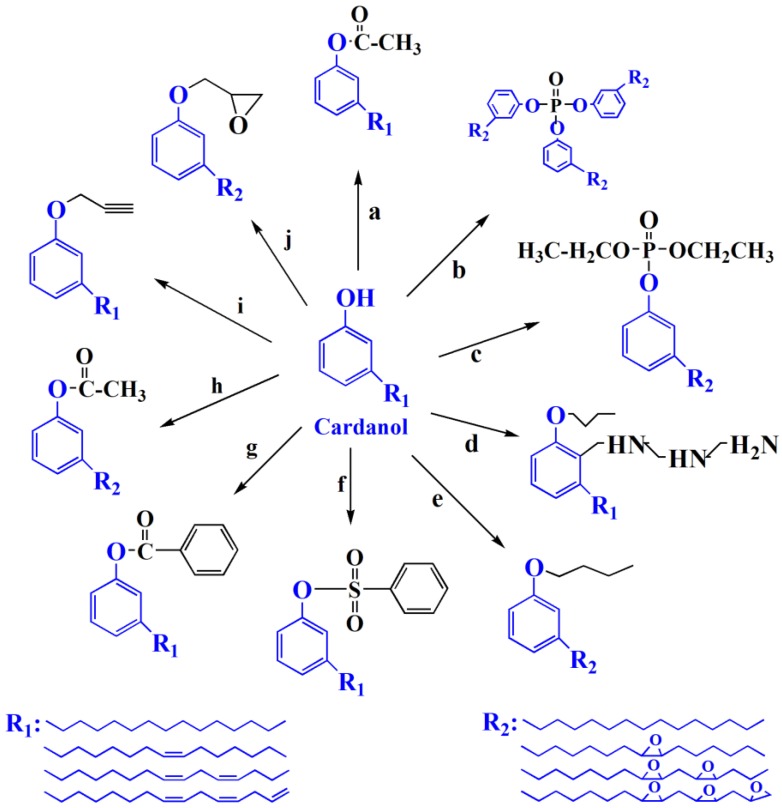
Chemical structure of cardanol based plasticizer.

**Figure 6:**
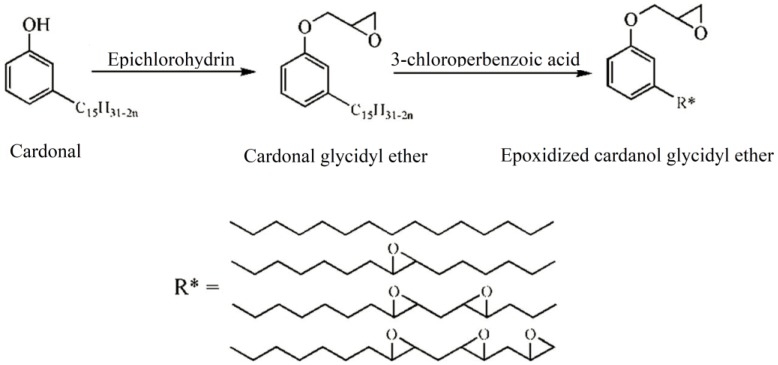
Synthesis of epoxidized cardanol glycidyl ether.

**Figure 7:**
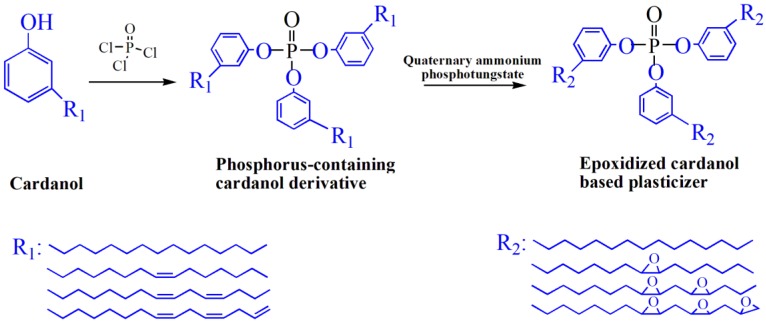
Synthesis routes of epoxidized cardanol based plasticizer (ECP).

Cardanol is an important chemical raw material, which has been widely used to prepare various plasticizers. Figure 5 shows the chemical structure of various of cardanol based plasticizers. Epoxidized cardanol glycidyl ether, as shown in Figure 5j and Figure 6, is synthesized from cardanol via substitution reaction and epoxidation reaction [33]. It is used to partly replaced DOP in soft PVC films. Epoxidized cardanol glycidyl ether improves flexibility and toughen soft PVC films and shows similar volatility, extraction and exudation resistance compared with DOP. Our group also synthesized a kind of epoxidized cardanol based plasticizer using quaternary ammonium phosphotungstate as catalyst [34]. Its chemical structure is shown in Figure 5b and Figure 7. Epoxidized cardanol-based plasticizers exhibit similar plasticizing efficiency with DOP. In addition, excellent solvent resistance and lower volatilization of epoxidized cardanol-based plasticizers maintain the long stability property of PVC products. The obtained cardanol-based plasticizer is biologically safe without acute toxicity, which makes it possible to be used in food packing and toys. 

### 2.2. Polyester Plasticizer and Macromolecular Plasticizer 

The polyester plasticizer is usually prepared by polycondensation of dibasic acid and diol. It is called “permanent plasticizer” because of its large molecular weight, better water resistance, excellent oil and solvent extraction and longer service life [38]. Polyester plasticizer also has the characteristics of low toxicity and safety. It is suitable for application in the fields with high health and safety requirements for plastic products such as beverage hoses, interior decoration, children’s toys, wire and cable. In addition, polyester plasticizers are especially suitable for surface finishing of floor tiles that are resistant to pollution, water or solvents due to its good durability. Traditional polyester plasticizers can be classified into adipic acid esters, sebacic acid esters and phthalic acid esters according to the type of dibasic acid structure. However, these polyester plasticizers are all derived from petrochemical resources and are against requirements of sustainable development. Renewable biodegradable polyester plasticizers have been paid more attention by industry and academia due to its good research and application prospects [39]. Moreover, compared with DOP, polyester plasticizers still have the disadvantages of poor plasticizing efficiency, poor processability and low temperature performance [40,41]. Lindström et al. examined the influence of molecular weight and branching on the performance of polyester [42]. They chose linear and branched poly(butylene adipate)s (PBA) to study the relationship between plasticizing effect and chemical structure. They prepared the samples with molecular weight ranging from 2000 to 10,000 g/mol, along with the content of branching agent between 0% and 1.8%. They concluded that the degree of branching is most decisive in plasticizing efficiency. They also investigated the migration of polyester plasticizer for PVC.

**Figure 8:**
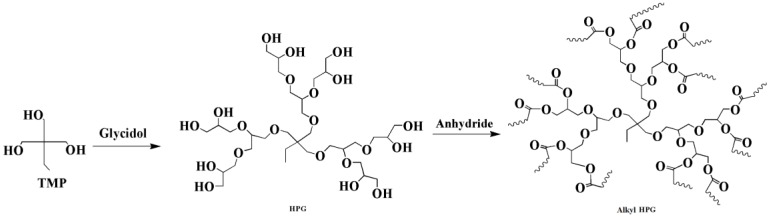
Synthesis of alkyl terminal hyperbranched polyglycerol (alkyl-HPG).

**Figure 9:**
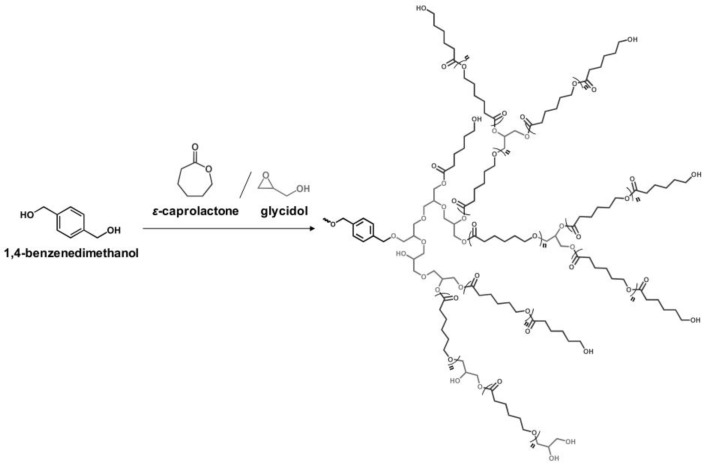
Synthesis of butyl-esterified highly branched polycaprolactone.

Glycidol can be derived from glycerol, which is a major compound generated during the production of biodiesel from the transesterification of plant oils and lipid-rich surplus streams from slaughterhouses and the rendering industry [43]. Recently, hyperbranched esters deriving from glycidol via a green one-pot process using neither toxic solvents nor expensive catalysts have been used to replaced DOP as main plasticizer [44,45]. Figure 8 and Figure 9 show the two kinds of hyperbranched esters: alkyl terminal hyperbranched polyglycerol (alkyl-HPG) and butyl-esterified highly branched polycaprolactone. PVC plasticized with alkyl-HPG presents enhanced transparency and thermal stability compared to PVC/DOP blends. Besides, alkyl-HPG plasticizer ia biologically safe without acute toxicity and has a lower degree of migration in leaching tests. Butyl-esterified highly branched poly(ε-caprolactone) (PCL) with various degree of polymerizations are prepared by ring-opening multibranching polymerization using glycidol as a single monomer and subsequent end-group modification, with structure of hyperbranched esters showing similar plasticizing effect on PVC and solvent extraction resistance as alkyl-HPG. In the case of the elongation at break, PVC plasticized with highly branched poly(ε-caprolactone)/glycidol copolymeric plasticizer shows the largest value of 397%, which is even greater than that of PVC/DEHP by 21%. 

Similar to epoxidized plasticizers, polyester plasticizers also serve as an alternative to phthalates because they may migrate from PVC products into human bodies or environment [46]. Materials such as long-chain linear polyester, poly(butylenes 2-methylsuccinate) (PBM) and aromatic hyperbranched polyesters (HBPEs) are considered as substitute of phthalates [47,48,49]. Sunny et al. studied the performances of the PVC cooperated with di-(2-ethyl hexyl) phthalate (DEHP), nitrile rubber (NBR), carboxylated nitrile rubber (XNBR) and epoxidized natural rubber (ENR) [50]. The results show that NBR enhanced the stability of the blends. When it comes to food degree PVC, the migration of plasticizers into food raises severe concerns [51,52]. 

Different starch sources and plasticizers influence films differently, especially the physical-chemical and mechanical properties. Zullo et al. prepared several types of plasticizers (glycerol, urea and formamide) and examined the influence of starch sources (maize, potato and wheat) on physical-chemical and mechanical properties of thermoplastic starch films [53]. The research shows that the composition of formamide and urea enhances physical-chemical and mechanical properties of thermoplastic starch films. Pushpadass et al. used native corn starch as raw material to produce 0.4–0.6 mm thick films [54]. Amylopectin and amylose account for 76.9% and 23.1% of native starch but the percentage only changes to 71.3–76.6% and 23.4–28.7%, respectively, during the extrudation process, which means that the degradation is not severe. 

**Figure 10:**
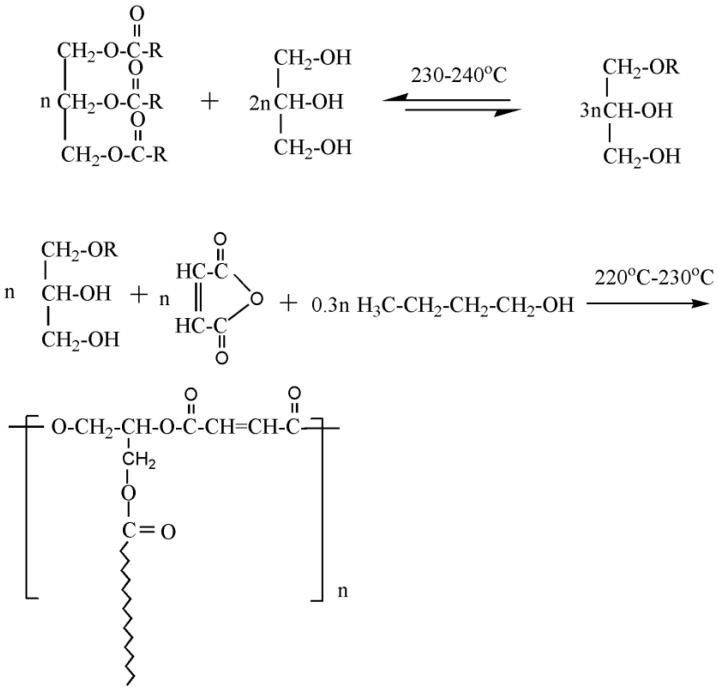
Synthesis bio-based polyester plasticizer from palm oil.

Diprotic acid for the synthesis of polyester plasticizers can be obtained by alcoholysis of vegetable oil. Figure 10 shows the synthesis route of bio-based polyester plasticizer from palm oil. As shown in Figure 10, palm oil monoglyceride (POM) is obtained by alcoholysis of palm oil [55]. Palm oil-based polyester plasticizer (POMP) is produced from POM and maleic anhydride via esterification. The obtained polyester plasticizer shows excellent compatibility with PVC and significantly improves thermal stability of PVC blends. When DOP is replaced by POMP gradually in the PVC blends, tensile strength decreases from 12.6 to 6.1 MPa, and the elongation at break decreased from 210.81% to 60.96%, illustrating that plasticizing effect of POMO is lower than DOP. This study provides general method for producing vegetable oil-based polyester plasticizer.

Fakhoury et al. pointed out that non-degradable materials damage the environment daily, thus bioplastics with the addition of glycerol or sorbitol are increasingly important [56]. The mechanical, physicochemical and physical properties of blends of manioc starch and gelatin are investigated during the process. As a result, glycerol and sorbitol, as plasticizers, increase the elongation at break and water vapor permeability (WVP) of manioc starch and gelatin blends. Moreover, sorbitol, poly(glutaric acid-glyceryl monooleate) (PGAGMO), poly(succinic acid-glyceryl monooleate) (PSAGMO) and PMMA are also modified and used as bio-based plasticizer of hydroxyl polymer [57,58,59].

### 2.3. Flame Retardant Plasticizer

Phosphate plasticizer is a functional plasticizer, which has both plasticizing effect and flame retardant effect on PVC products. Phosphate esters have good compatibility with various resins and synthetic rubber such as PVC, cellulose, polyethylene (PE) and polystyrene (PS). They have been used as cellulose plasticizers for more than 100 years. With the high density of urban construction and the rapid development of transportation, the flame-retardant performance requirements of various products are increasing. Phosphate ester plasticizers have been widely used, especially in fireproof and non-flammable products such as the preparation of rubber, plastics, military products, textiles, electrical appliances, conveyor belts and various building materials. The largest producer and consumer country of flame retardants is the United States. Phosphorus-based flame retardants in China have a large gap compared with the United States in terms of production capacity, output and variety. The proportion of phosphorus-based flame retardants in Chinese plastic additives is low. Therefore, adjusting the product structure of plasticizers and flame retardants, and increasing the proportion are essential conditions for the development of phosphate ester plasticizers in the future.

Phosphate plasticizer is the main plasticizer for PVC and has the following characteristics: good flame-retardant performance, better compatibility, good mold resistance, poor weather resistance, high toxicity and high price. Phosphate esters are normally prepared by esterification of phosphorus oxychloride or phosphorus trichloride with an alcohol or a phenol. Commonly used phosphate esters are triethyl phosphate, tributyl phosphate, *o*-crepe phosphate, tri-*p*-tolyl phosphate, triphenyl phosphate, and xylene diphenyl phosphate [60,61]. Phosphate esters are usually used as flame retardant plasticizers, especially in PVC products, because PVC products will not present self-extinguishing when the PVC products contain less than 20 wt % of phosphate plasticizer [62]. Phosphate esters are also used to improve flame retardancy of epoxy resins combined with nitrogen, silicon or boron [63,64,65,66]. There are many kinds of phosphate type plasticizers: tributyl phosphate (TBP), tris(2-chloroethyl) phosphate (TCEP), triphenyl phosphate (TPP), tris(2-butoxyethyl) phosphate (TBEP), tris(2-ethylhexyl) phosphate (TEHP), tricresyl phosphate (TCP), tris(2-chloro-isopropyl) phosphate (TCPP), tris(1,3-dichloroisopropyl) phosphate (TDCP), and triaryl phosphates [67,68].

**Figure 11:**
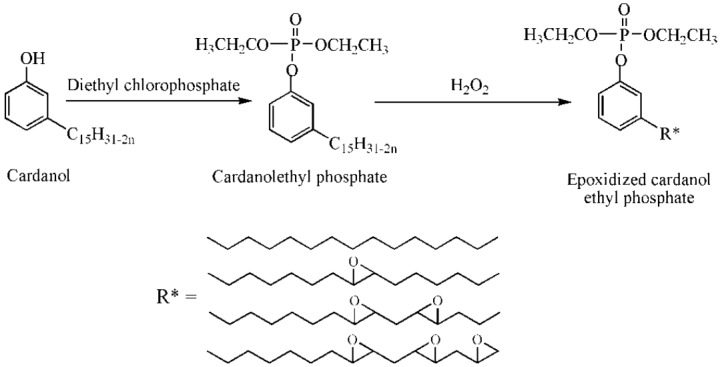
Synthesis of epoxidized cardanol ethyl phosphate.

Epoxidized cardanol diethyl phosphate is a kind of phosphate esters, is synthesized from cardanol and enhances thermal stability of PVC films. Figure 5c and Figure 11 show its chemical structure and synthesis route. Glass transition temperature (*T*_g_) of PVC films containing 40 wt % of epoxidized cardanol diethyl phosphate reach 33.20 °C [69].

**Figure 12:**
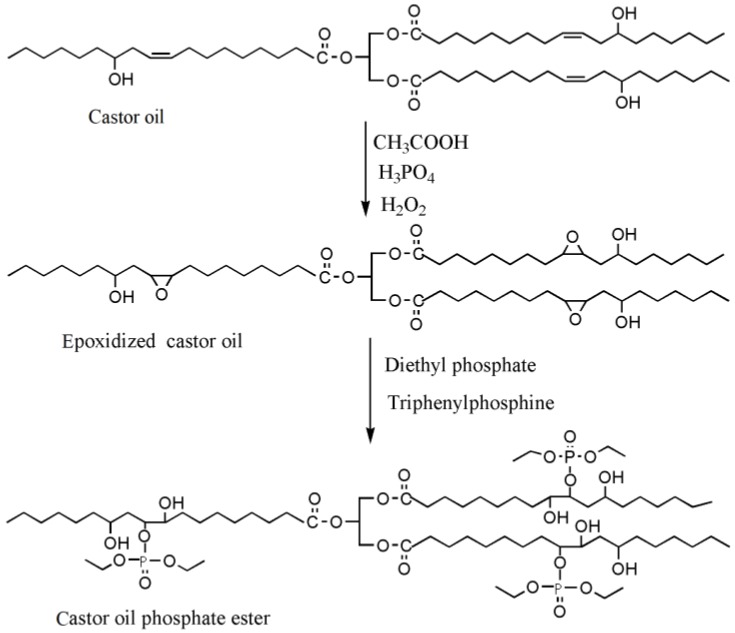
Synthesis of castor oil phosphate ester.

Feng et al. used castor oil as basis to synthesize another novel flame-retardant plasticizer based on castor oil (FRC), as shown in Table 1a. They prepared FRC by a three-step procedure of alcoholysis, epoxidation and ring opening reaction and used molding machine to obtain the blends of PVC and FRC [70]. The addition of FRC reduces torque by 33.6% and enhances the flame-retardant properties. LOI js enhanced by 31.9%. The addition of FRC causes a significant decrease of 61.94% in tensile strength and approximately 60% in elongation at break for PVC materials, which indicates that FRC has plasticizing effect on PVC. Our group synthesized a series of flame-retardant plasticizers based on vegetable oil [71,72,73,74,75,76]. A flame-retardant plasticizer based on castor oil (PPC) is synthesized by castor oil, H_2_O_2_, phosphate and diethyl phosphate [71]. Figure 12 and Table 1b show its chemical structure and synthesis route. The results show that the glass transition temperature increases from 26.5 to 52.6 °C with the content of PPC in 0 to 20 g in 100 g of PVC while the thermal stability is maintained by PPC until 350 °C. In addition, the leaching test indicates that solvent extraction resistance of PPC is better than DOP.

**Figure 13:**
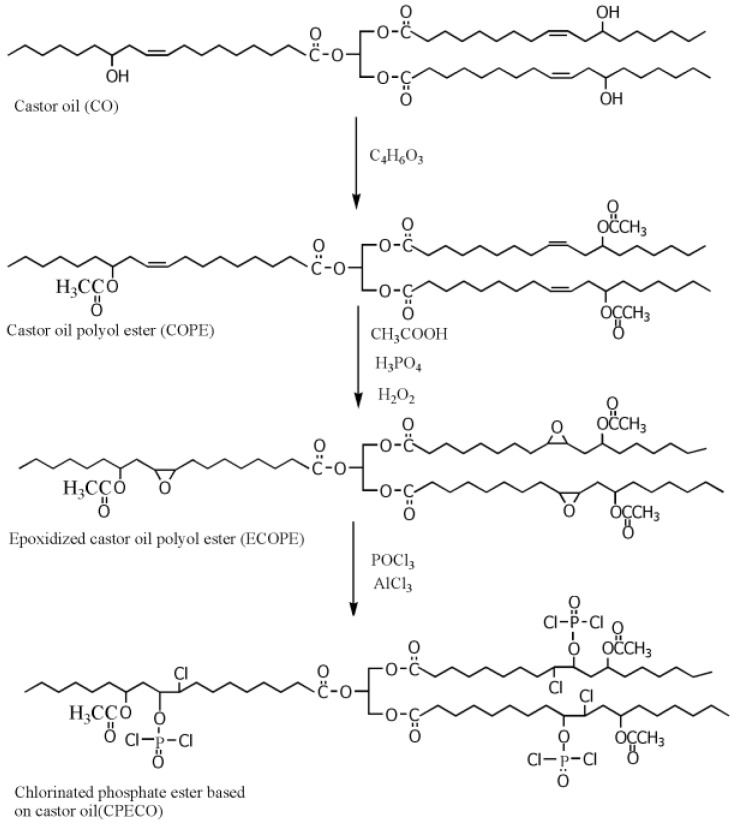
Synthesis of chlorinated phosphate ester based on castor oil.

Chlorine and phosphorus containing vegetable-based plasticizer is also synthesized from castor oil, as shown in Figure 13 and Table 1c [72]. LOI of PVC containing 40 wt % of DOP is 23.6%. When half the DOP is replaced with chlorinated phosphate ester based on castor oil, LOI of PVC blends reaches 35.4%, peak heat release (pHRR) decreases from 379.00 to 289.00 kW/m^2^, and total heat release (THR) decreases from 31.78 to 19.12 MJ/m^2^. 

**Figure 14:**
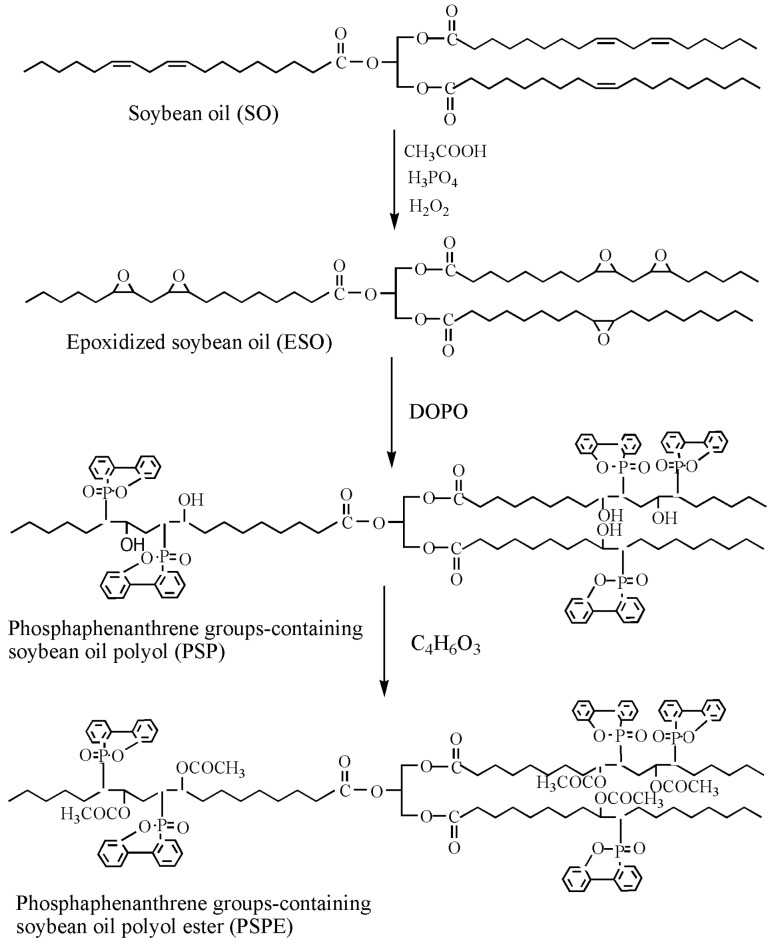
Synthesis of DOPO groups-containing soybean oil polyol ester.

**Figure 15:**
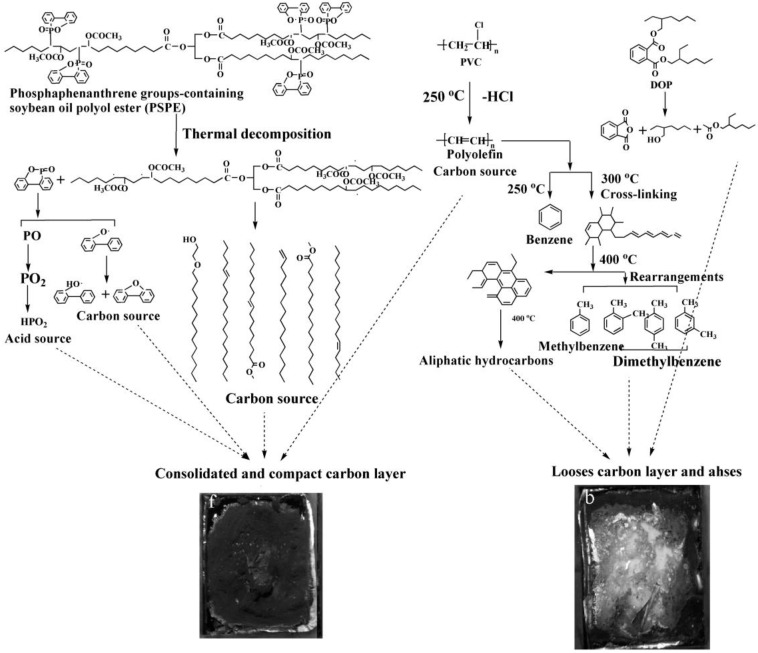
Flame retardant mechanism of DOPO groups-containing soybean oil polyol ester.

Flame-retardant plasticizer based on soybean oil containing phosphaphenanthrene groups (PSPE) is also prepared [73], as shown in Figure 14 and Table 1d shows its chemical structure. The results show that LOI of PVC blends plasticized with 40d. The results show that PVC blends with PSPE possess better flame retardancy and higher thermal stability, and the LOI value reaches 36.25. PSPE releases PO_2_, HPO_2_ and long fatty acid chains during burning. As shown in Figure 15, groups containing soybean oil polyol ester have flame-retardant effect on PVC blends by promoting formation of char residue. Phosphaphenanthrene groups-containing soybean oil-based plasticizer (PSOPE) is prepared [74]. Table 1e shows its chemical structure. The results show that LOI of PVC blends plasticized with 40 wt % of PSOPE reaches 36.2%, indicating that flame-retardant performance of PSOPE is similar to PSPE.

**Figure 16:**
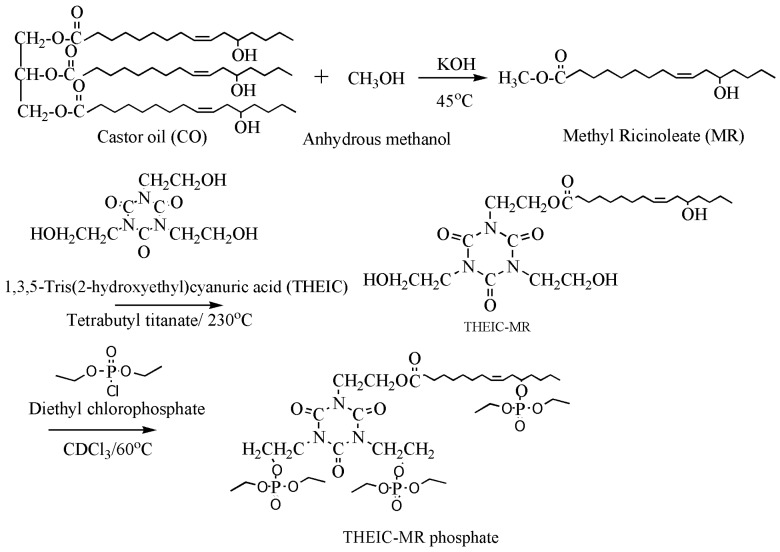
Synthesis of THEIC-MA phosphate.

Synergistic flame retardance can be obtained by combining nitrogen, phosphorus and chlorine in one molecular structure of vegetable oil-based flame-retardant plasticizer [75,76]. Table 1f,g shows the chemical structure of synergistic flame retardant plasticizer based on vegetable oil. Figure 16 shows the synthesis route of 1,3,5-tris(2-hydroxyethyl)cyanuric acid groups and diethyl phosphate groups containing castor oil-based plasticizer (THEIC-MA phosphate). THEIC-MR-phosphate enhances the flame-retardant performances of PVC blends by promoting the formation of char residue in solid phase, and dilution and isolation of oxygen in gas phase. Cone tests show that the time to ignition increases, combined with the decrease of heat release rate (HRR) value, indicating that THEIC-MR-phosphate effectively improves flame-retardant performance of PVC materials and delays fire process.

**Figure 17:**
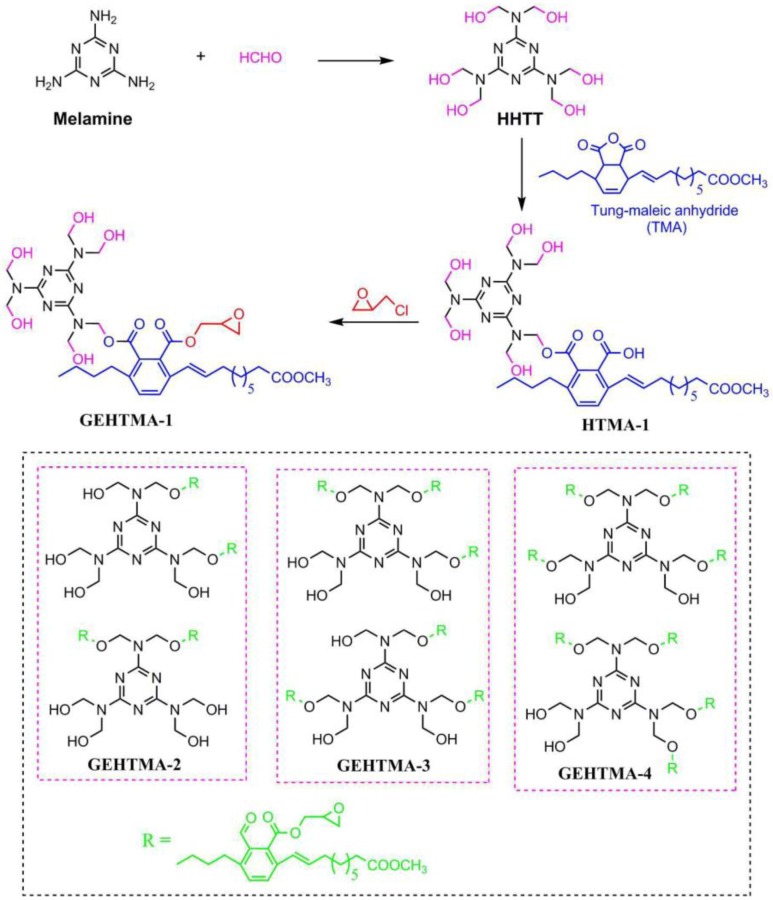
Synthesis of hydroxyl and nitrogen rich group-containing tung-oil-based ester plasticizers.

Hydroxyl and nitrogen rich group-containing tung oil-based ester (GEHTMA-1, GEHTMA-2, GEHTMA-3 and GEHTMA-4) plasticizers are prepared and used to replace dioctyl terephthalate (DOTP) [77]. Figure 17 and Table 1h,i,j show synthesis route and their chemical structure. GEHTMA-3 displays better mechanical properties and endows PVC resins with well-balanced properties of flexibility and strength, which are attributed to simultaneous introduction of hydroxyl, epoxy, benzene ring, ester and nitrogen rich groups into GEHTMA-3 structures.

**Figure 18:**
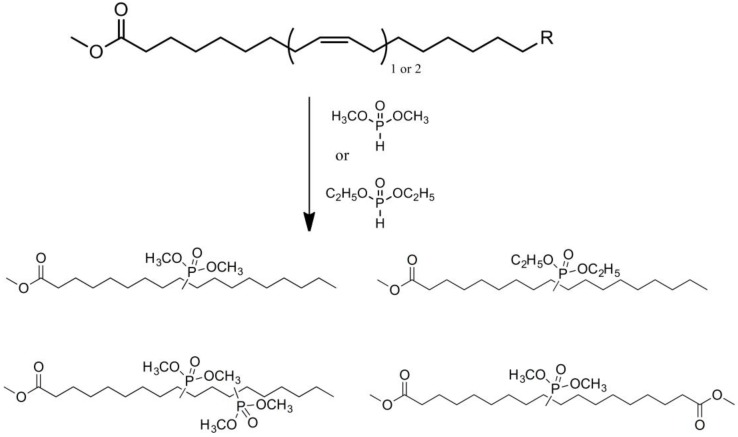
Synthesis of phosphonate groups containing bio-based plasticizers.

Lapinte et al. used methyl oleate, methyl linoleate, and oleic diacid as raw materials to prepare a series of flame-retardant plasticizers: dimethyl (methyl oleate)phosphonate (PMO), diethyl (methyl oleate)phosphonate (PMO2), dimethyl (methyl linoleate)phosphonate (PML), and dimethyl (dimethyl oleate)phosphonate (PDE) [75] (Figure 18 and Table 1k,l,m,n). PVC materials plasticized with these flame-retardant plasticizers show similar thermal properties compared with diisononyl phthalate blends and excellent flame-retardant performances. Phosphonate groups promote the formation of a char layer, protecting the material against fire. 

### 2.4. Citric Acid Ester Plasticizer

Citric acid ester plasticizers are important, environmentally-friendly plasticizers because of their safety, non-toxicity and precipitation resistance. They have been approved in the United States, the European Union and other developed countries to be used in plastic products in close contact with the human body and meet high hygiene requirements such as for food packing, children’s toys, medical equipment and sanitary products. Therefore, citrate plasticizers have become the first choice for safe and non-toxic plasticizer products in the plastics industry globally. There are more than 50 kinds of citric acid plasticizers, and about 15 kinds of products have been produced at large-scale by the industrialized manufacturing industry. Acetyl tributyl citrate (ATBC) and tributyl citrate (TBC) have been studied in-depth and industrialized due to excellent performances. ATBC has good water and light resistance, good thermal stability without color, and maintains good flexibility at low temperatures. TBC has good compatibility with cellulose resin, PVC, PP, and its plasticizing effect is remarkable. In addition, TBC has antibacterial and flame-retardant properties, which further expand its applicability. However, the price of the plasticizer is relatively high, so it is mainly used in fields with high requirements for non-toxic safety of products such as food packaging materials, children’s toys, medical equipment and packaging.

Citric acid has been commercially produced through large-scale fermentation, thus undoubtedly is considered a raw material for producing plasticizers for PLA, PVC and other materials. Shi et al. prepared a novel plasticizer named glycerol co-plasticized thermoplastic starch (CGTPS) by melting and blending citric acid (CA) [79]. The results show that esterification occurs when the content of CA increases. In comparison with commonly used glycerol plasticized thermoplastic starch (GTPS), the CGPTS shows partial esterification and lower molecular weight. Moreover, the interaction between plasticizers and starch becomes stronger, thus resisting retrogradation and enhancing its mechanical properties. According to Chabrat et al., the presence of citric acid has positive effects on mechanical and morphological properties of blends made from wheat flour and glycerol [80]. They claimed that citric acid functioned as compatibilizer, enhancing blend compatibilization. Schilling et al. studied the suitability of citric acid (CA) for Eudragit RS PO system [81]. The results show that the monohydrate form apparently benefits the extrusion of Eudragit RS PO, while the anhydrous form is less effective. Flexibility of Eudragit RS PO films increases with the addition of CA monohydrate (CAMH). Thus, CAMH serves as a great assistant for Eudragit RS PO system. Olivato et al. obtained starch/poly films by using maleic anhydride (MA) and CA [82]. The results show that CA enhances esterification/transesterification reactions better than MA and more CA can increase the elongation at break of films. In addition, this process produces films that may become the substitute of non-biodegradable plastics. Shi et al. examined the influence of CA on swelling degree, crystallinity and cytotoxicity of PVA/starch films [83]. FT-IR spectroscopy shows esterification between CA and starch occurs at 140 °C; esterification is much easier between PVA and CA. The tensile strength of the film reaches its maximum when the content of CA is 30 wt % of PVA/starch films. Besides, the results show that there is no apparent toxicity on cells when the content of CA is less than 20 wt % of PVA/starch films. Crosslinking is another precious character of citric acid. Seligra et al. took CA as crosslinking agent to produce starch–glycerol films, which are biodegradable and non-retrogradable [84]. The obtained films remain amorphous for more than 45 days. The method avoids retrogradation of starch–glycerol films and maintains the degradability. Garcia et al. changed amounts of critic acid to examine the crosslinking effect [85]. The calculation results of water vapor permeability (WVP) show that the increase of content of citric acid would reduce the diffusion coefficient and WVP. In other words, such reduction apparently suggests the occurrence of crosslinking. Thus, crosslinking is an effective approach to improve the properties of starch products. 

Zuraida et al. used citric acid and water as secondary additive to prepare bio-plastic starch (BPS). FT-IR results show that the addition of CA at 30 wt %, intensifies the properties of BPS to the degree 40% higher than that of BPS with additional water [86] because the addition of CA disrupts molecular hydrogen bond effectively and avoids the appearance of poor starch chain samples with cavities. Ghanbarzadeh et al. studied the effect of CA on corn starch-based edible films [87]. The results show that CA increases the water vapor barrier property (WVBP) and the ultimate tensile strength (UTS) of corn starch-based edible films. However, when the percentage of CA increases from 10 to 20 wt %, the UTS decreases from 6.57 to 1.80 MPa. Thus, triethyl citrate plasticizer can be used to prepare “Green” films, which possess better intercalation and an exfoliated structure [88].

### 2.5. Glyceryl Ester Plasticizer

**Figure 19:**
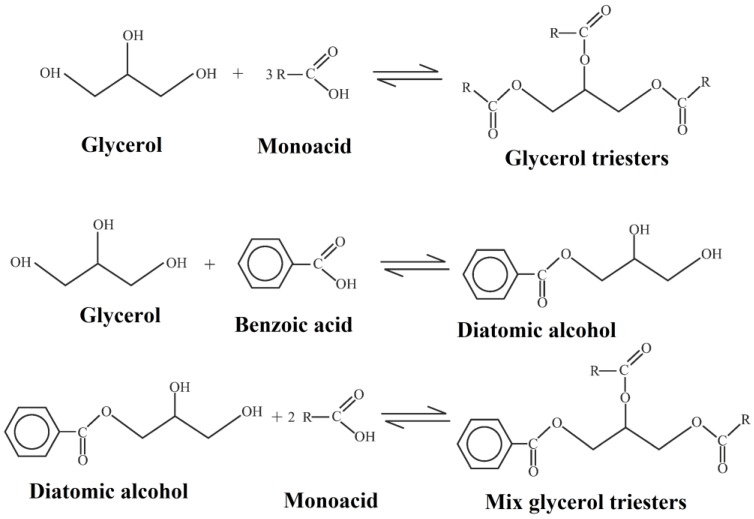
Synthesis of glycerol triesters and mix glycerol triesters.

Glycerol, as a by-product in biodiesel production, is widely applied in microbial production, biomedical applications and plasticizers [89,90,91,92]. Palacios et al. synthesized glycerol-based plasticizers and applied them as an alternative of phthalates [93]. They prepared ight kinds of glycerol triesters from butanoic (butyric), propanoic, isobutanoic (isobutyric), isopentanoic (isovaleric), and benzoic acids. Figure 19 shows their synthesis routes. These low-molecular-weight glycerol triesters are all compatible with PVC and have effective plasticizing effect on PVC. Sahu et al. studied the biodegradation of rosin-glycerol ester derivative [94]. They explored the biodegradation mechanism of rosin glycerol ester and applied it as a biodegradable material. Thomazine et al. found that the addition of glyceryl ester enhances the elongation at break and water vapor permeability (WVP) of gelatin blend [95]. McHugh et al. also reported that sorbitol and glycerol together significantly reduce tensile strength, intensifying elongation of protein edible films [96]. Glyceryl ester is also an ideal addition for starch plasticizers [97,98,99]. Chang et al. prepared glycerol plasticized wheat starch (GPS)/CN nanocomposite through a casting process [99]. As the content of such nanoparticles is increased from 0 to 5 wt %, the tensile strength also increases from 3.15 to 10.98 MPa. The effect of glyceryl ester on performance of thermoplastic starch (TPS) blends is investigated; the results illustrate that the shear viscosity of thermoplastic starch (TPS) blends decreases by 20% when the percentage of glycerol increases from 36% to 40% [100,101].

### 2.6. Other Plasticizers 

**Figure 20:**
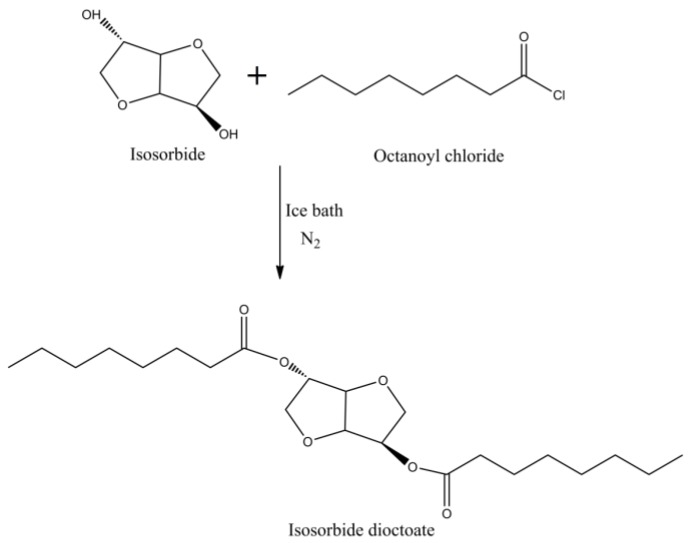
Synthesis of isosorbide dioctoate.

Apart from plasticizers mentioned above, there are also other bio-based plasticizers such as glucose ester, traffic waste oil, isosorbide, ricinoleic acid and cardanol [101,102,103,104,105,106]. Yin et al. prepared glucose from liquefaction of cellulose and utilized laser desorption ionization–mass spectrometry (LDI-MS) method to evaluate the performance [107]. The results show that glucose esters have great miscibility with PVC and the maximum elongation at break is reached when the content of glucose ester is 40 wt %. Cataldo et al. claimed that biodiesel can be used as an effective low-temperature plasticizer, which can be applied in winter tires [108]. Isosorbide dioctoate as plasticizer for PLA presents a better miscibility than dioctyl terephthalate. Figure 20 shows the synthesis of isosorbide dioctoate. The light transmittance reduces when the content of isosorbide dioctoate increases in PLA blends but still shows high transparency. The plasticizing performances indicates that isosorbide dioctoate has great potential to replace traditional phthalate plasticizers [109]. The effect of oligo(isosorbide adipate) (OSA), oligo(isosorbide suberate) (OSS) and isosorbide dihexanoate (SDH) on PVC plasticizers are investigated; the blends plasticized with SDH are similar to DIOP blends, while a film plasticized by OSA or OSS exhibits higher glass transition temperature [110]. Thus, these synthesized isosorbide plasticizers serve as potential substitutes of DOP.

More and more plasticizer products derived from cardanol, a renewable main by-product of the cashew industry have been applied in manufacturing [111]. Greco et al. did many excellent works on producing various of bio-based plasticizers using cardanol as raw material. They studied the effect of cardanol based plasticizers on PVC by scanning the miscibility [112]. The results show that partial miscibility occurs between PVC and cardanol acetate, as shown in Figure 5a, while epoxidized cardanol acetate (Figure 7h) leads to complete miscibility with PVC. Therefore, cardanol acetate is used as second plasticizer to replace toxic phthalate plasticizers. Greco also studied the mechanical, solubility and durability properties of cardanol-derived plasticizers and they exhibit better performance [113,114]. Besides, he also found that cardanol derivatives are innovative plasticizers for poly(lactic acid) (PLA) and claimed that the suitability of cardanol based plasticizers would be wider in the future [115,116]. Apart from PLA or PVC, cardanol is widely used for alumina/toluene tape casting slip and silica-filled natural rubber [117,118].

**Figure 21:**
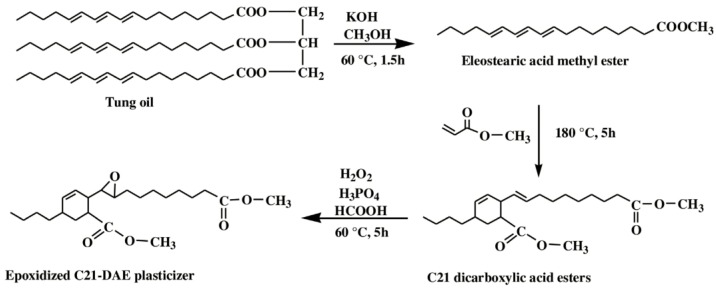
Synthesis of C21 dicarboxylic acid ester.

Li et al. used a tung oil-derived epoxidized dicarboxylic acid dimethyl ester (C21 dicarboxylic acid ester) as plasticizer and auxiliary stabilizer for PVC [119]. Figure 21 shows its synthesis route. It is synthesized from tung oil via transesterification, Diels–Alder reaction and epoxidation. PVC blends plasticized with C21 dicarboxylic acid ester as main plasticizer and auxiliary stabilizer show excellent thermal stability and plasticizing performance, combined with improved migration and volatility stabilities compared to dioctyl terephthalate (DOTP) samples.

**Figure 22:**
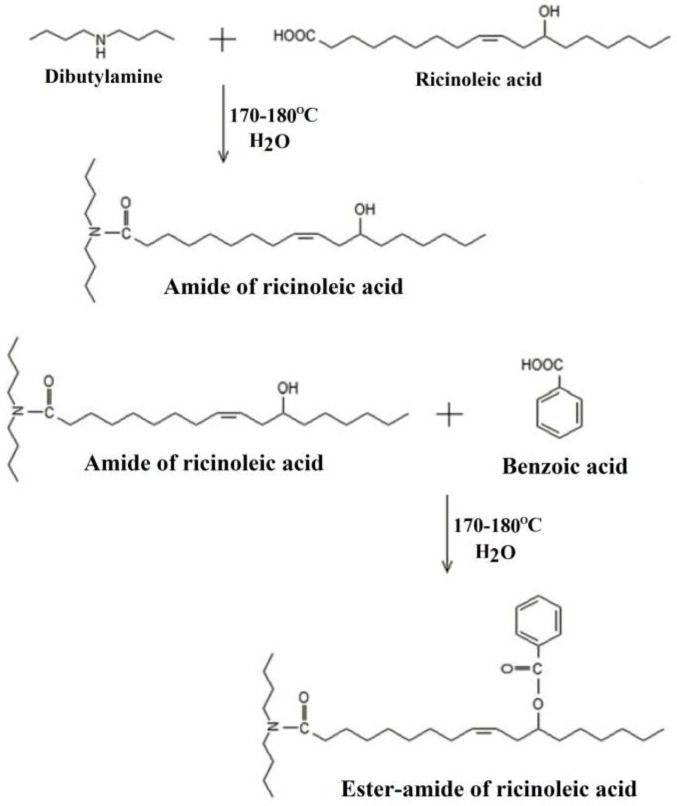
Synthesis of ester-amide of ricinoleic acid.

Vegetable fatty acids such as ricinoleic acid are also used to produce bio-based plasticizers. Ester-amide of ricinoleic acid, as shown in Figure 22, is synthesized and used as primary plasticizer to replace phthalate plasticizers in PVC materials [105]. *T*_g_ of PVC materials blended with 40 wt % of ester-amide of ricinoleic acid reaches −13.5 °C. 

**Figure 23:**
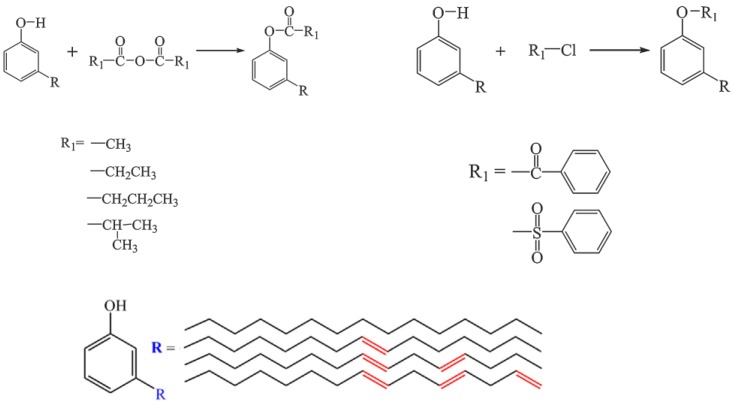
Synthesis of cardanol esters.

A series of cardanol-based plasticizers were produced by Yang group [111]. The chemical structure and synthesis route of cardanol esters are shown in Figure 5a,f,g,h and Figure 23. These caedanol esters decrease *T*_g_ of PVC and show excellent biodegradability and migration stability. Biodegradability of cardanol esters are better than cardanol, which provides potential methods to solve environmental problems produced by non-degradability of cardanol. 

**Figure 24:**
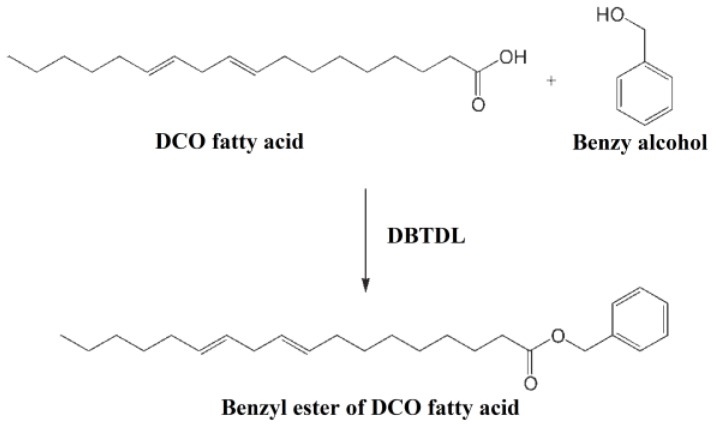
Synthesis of benzyl ester of DCO fatty acid.

Esterification reaction of vegetable oil fatty acid and benzyl alcohol is used to synthesize bio-based plasticizers (benzyl ester of DCO fatty acid) [120]. Figure 24 shows synthesis route of benzyl ester of DCO fatty acid (BE), which is used as auxiliary plasticizer to prepare soft PVC materials. Tensile strength, percentage elongation and thermal degradation properties are improved after blending BE in PVC formulations. Good anti-leaching properties and plasticizing performances of BE make it an environmentally-friendly plasticizer.

**Figure 25:**
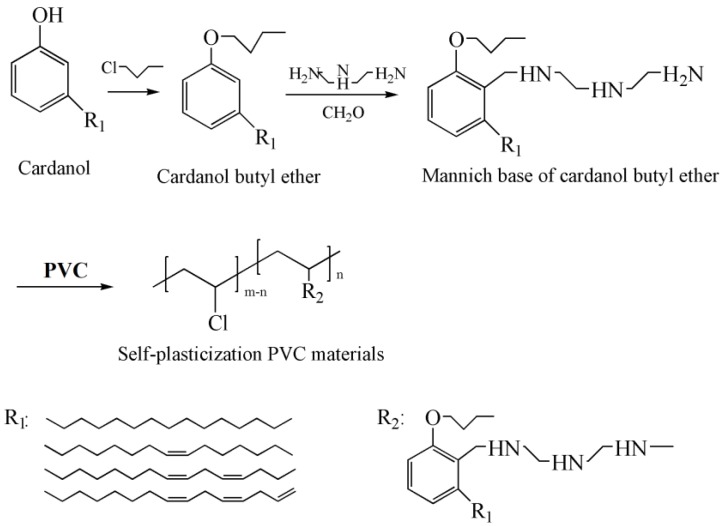
Synthesis of self-plasticization PVC via substitution reaction with minnich base of cardanol butyl ether.

Internal plasticizer is the part of polymer that has plasticizing effect on polymer by increasing distance of polymer chains, decreasing the interaction force of main chains of polymer and increasing the possibility of mutual movement of polymer chains [121,122]. Self-plasticization PVC via substitution reaction with minnich base of cardanol butyl ether is obtained, and shown in Figure 25 [123]. Minnich base of cardanol butyl ether, as shown in Figure 5d, is internal plasticizer in this study. *T*_g_ of self-plasticization PVC deceases from 85.6 °C to 49.3 °C. The decrease of tensile strength and increase of elongation at break for self-plasticization PVC illustrates the internal plasticizing effect. Nonmigrating plasticized PVC plasticized with mannich base of waste cooking oil methyl ester is also investigated via the same method [124]. No plasticizer loss for self-plasticization PVC materials in leaching tests gives these materials potential to be used in food packaging and toys.

**Figure 26:**
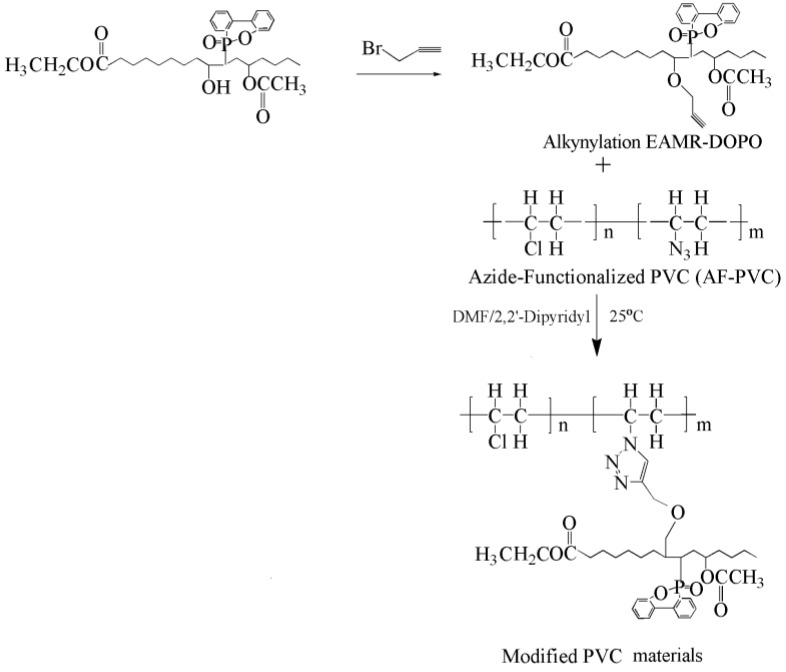
Synthesis internally plasticized PVC with alkynylation EAMR-DOPO.

**Figure 27:**
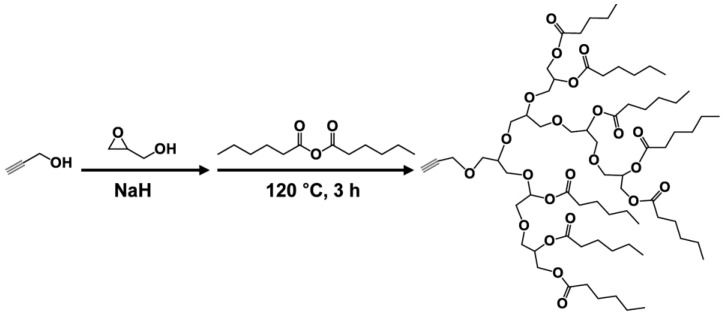
Synthesis of hexyl-terminated hyperbranched polyglycerol.

Click reaction of azide functionalized PVC and alkynyl group containing bio-based derivatives produces internally plasticized PVC materials [125,126,127,128]. Alkynylation EAMR-DOPO (Figure 26), propargyl ether cardanol (Figure 5i), and hexyl-terminated hyperbranched polyglycerol (Figure 27) are grafted onto PVC chain. The obtained PVC materials show lower *T*_g_ and improved flexibility compared to PVC. No plasticizer migration is found in leaching tests. The excellent solvent extraction resistance and volatility resistance give these internally plasticized PVC materials potential for applications in products with a high migration resistance requirement. 

## 3. Conclusions

Biomass resources have been frequently used to produce PVC plasticizers as low-cost raw materials. Bio-based plasticizers designed for different application are mostly based on chemical structure of biomass resources. Cardanol is a promising alternative to traditional petrochemical-based *o*-phthalate plasticizers due to its benzene ring structure and active hydroxyl group. Vegetable oil contains flexible long fatty acid chains and rich unsaturated bonds and can be used to produce epoxy plasticizer. Epoxidized soybean oil (ESO) has been industrialized and used as primary plasticizer in food packing materials. Vegetable oil-based flame-retardant plasticizers provide rich carbon combined with flame-retardant elements to improve flame retardancy of PVC product by promoting the formation of char residue. Cardanol and vegetable oil have been the two most important biomass feedstocks for producing plasticizers. Internally plasticized strategy produces excellent polymer materials with plasticity and flexibility but without plasticizer migration. Thermal stability, migration stability, biodegradability and plasticizing efficiency of bio-based plasticizers have been widely investigated and discussed in the literature. However, research on biological toxicity of bio-based plasticizers on human body and animal such as experimental mice and fish is scant. From an industrialization point of view, biological toxicity of bio-based plasticizers should be taken into account. Considerable efforts should be devoted to investigating the relationship between biological toxicity and chemical structure of bio-based plasticizers. Then, we can design non-toxic and environmentally-friendly biomass-based plasticizers to replace reproductive toxicity of o-phthalate plasticizers. Biomass raw materials, synthesis strategy, performances of PVC materials plasticized with bio-based plasticizers, and recent development in plasticizer field, as discussed in this article, might be used for the commercialization of bio-based, low-cost, environmentally-friendly plasticizers for numerous applications.

## Figures and Tables

**Table 1:** Flame-retardant elements (phosphate, chlorine and nitrogen) containing vegetable oil-based plasticizers.

Flame Retardant Elements (Phosphate, Chlorine and Nitrogen) Containing Vegetable Oil Based Plasticizers	References	No.
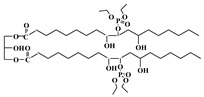	[70]	a
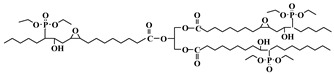	[71]	b
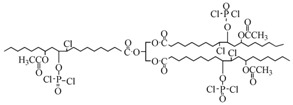	[72]	c
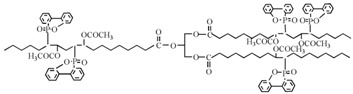	[73]	d
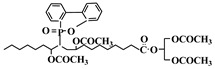	[74]	e
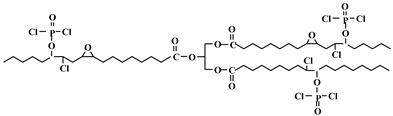	[75]	f
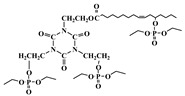	[76]	g
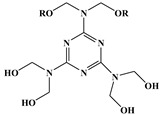	[77]	h
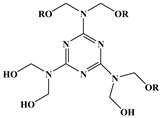	[77]	i
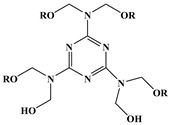	[77]	j
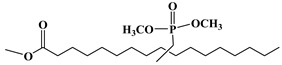	[78]	k
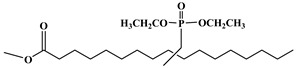	[78]	l
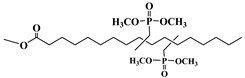	[78]	m
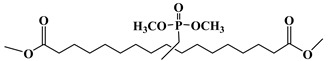	[78]	n
